# The King's Coroner

**Published:** 1906-09

**Authors:** 


**The King's Coroner.** By R. Henslowe Wellington. Vol. II. Pp. xvi., 148. London: Baillière, Tindall and Cox, 1906.

This book is a sequel to a previous work by the same author, comprising a collection of the statutes relating to the office of the King's coroner.

The present volume, after giving a slight introductory sketch of the history of the office of coroner, is confined to the practice and procedure in the Coroner's Court, and claims to be complete in itself. To justify that claim the book should, however, have contained the text of the Coroner's Act, 1887, and of the amending Act of 1892, as questions are bound to arise in practice which necessitate a reference to the text of the Act.

Of course the present volume is mainly of interest to the legal profession.

As, however, any doctor is liable to find himself in the position of having to attend and give evidence at an inquest, it is well that he should have available a book which will give him a clear and concise idea of what the procedure of the court is, and the present volume answers this purpose. It touches upon those points which are of direct interest to the medical profession, such as the cases which should be reported to the coroner, the position of medical witnesses, the occasions when a *post-mortem* examination is necessary, and the payment of medical fees, and it gives the general rules which govern the admissibility of evidence.

The book does not profess to deal with the principles relating to the matters which form the subject of inquiry, but as a handbook of the practice of the court it appears to be clear and adequate.

